# Implementation of evidence-based practices in normal delivery care

**DOI:** 10.1590/1518-8345.2177.2988

**Published:** 2018-03-08

**Authors:** Clodoaldo Tentes Côrtes, Sonia Maria Junqueira Vasconcellos de Oliveira, Rafael Cleison Silva dos Santos, Adriana Amorim Francisco, Maria Luiza Gonzalez Riesco, Gilceria Tochika Shimoda

**Affiliations:** 1 Doctoral student, Escola de Enfermagem, Universidade de São Paulo, São Paulo, SP, Brazil. Assistant Professor, Departamento de Ciências Biológicas e da Saúde, Universidade Federal do Amapá, Macapá, Amapá, Brazil.; Universidade Federal do Amapá, Departamento de Ciências Biológicas e da Saúde, Universidade Federal do Amapá, Macapá, Amapá, Brazil; 2 PhD, Associate Professor, Escola de Enfermagem, Universidade de São Paulo, São Paulo, SP, Brazil.; 3 PhD, Adjunct Professor, Departamento de Ciências Biológicas e da Saúde, Universidade Federal do Amapá, Macapá, AP, Brazil.; 4 PhD, Professor, Escola de Enfermagem, Universidade de São Paulo, São Paulo, SP, Brazil.; 5 PhD, RN, Hospital Universitário, Universidade de São Paulo, São Paulo, SP, Brazil.

**Keywords:** Labor Obstetric, Natural Childbirth, Evidence-Based Practice, Translational Medical Research, Clinical Audit, Obstetric Nursing

## Abstract

**Objective:**

to evaluate the impact of the implementation of evidence-based practices on normal delivery care.

**Method:**

quasi-experimental, before-and-after intervention study conducted in a public maternity hospital, Amapá. Forty-two professionals and 280 puerperal women were interviewed and data from 555 medical records were analyzed. The study was developed in three phases: baseline audit (phase 1), educational intervention (phase 2) and post-intervention audit (phase 3).

**Results:**

after the intervention, there was an increase of 5.3 percentage points (p.p.) in the normal delivery rate. Interviews with the women revealed a significant increase of the presence of companions during labor (10.0 p.p.) and of adoption of the upright or squatting position (31.4 p.p.); significant reduction of amniotomy (16.8 p.p.), lithotomy position (24.3 p.p.), and intravenous oxytocin (17.1 p.p.). From the professionals’ perspective, there was a statistical reduction in the prescription/administration of oxytocin (29.6 p.p.). In the analysis of medical records, a significant reduction in the rate of amniotomy (29.5 p.p.) and lithotomy position (1.5 p.p.) was observed; the rate of adoption of the upright or squatting position presented a statistical increase of 2.2 p.p.

**Conclusions:**

there was a positive impact of the educational intervention on the improvement of parturition assistance, but the implementation process was not completely successful in the adoption of scientific evidence in normal delivery care in this institution.

## Introduction

The predominant model of childbirth care in Brazil is characterized by the abusive or inappropriate use of interventions (routine amniotomy, lithotomy and intravenous infusion of oxytocin) and restriction of the parturients’ rights (restriction of the presence of companions) in all stages of labor. These problems can be prevented or reduced by adopting the best scientific evidence available in obstetric care^1^.

A hospital-based study called Birth in Brazil, coordinated by the National School of Public Health Sergio Arouca, Oswaldo Cruz Foundation (Rio de Janeiro), surveyed the profile of obstetric care in the country through an investigation of 23,940 puerperae and pointed out that some practices, such as lithotomy position (91.7%), oxytocin (36.4%), amniotomy (39.1%), Kristeller maneuver (36.1%), and cesarean sections (51.9%) continue to be offered as routine care in normal-risk pregnancies^2^.

If, on the one hand, the advance of modern obstetrics has contributed to the improvement of indicators of maternal and perinatal morbidity and mortality, on the other hand, it has allowed the implementation of a model that deals with pregnancy and childbirth as if they were diseases rather than as expressions of health. As a consequence, women and newborns (NBs) are exposed to high intervention rates^1^.

Therefore, the World Health Organization (WHO) proposed in 1996 some changes in labor and delivery care, including the need to rescue the idea of childbirth as a natural event, with a stimulus for the work of obstetric nurses, the use of practices based on the best scientific evidence and access to appropriate technologies for childbirth care^3^. In this perspective, the importance of health policies and practices to be grounded on the best available evidence and of translating knowledge into action, promoting the effectiveness and safety of interventions, stands out^4^ ^-^ ^6^.

However, introducing practices scientific evidence-based care practices requires more than knowledge and beliefs, since changes of behavior, overcoming barriers and filling gaps in the transfer of knowledge are required^4^.

Implementation refers to the use of evidence in clinical practice, through changes in care and/or in health services^4^, and clinical audit is one of the strategies to achieve its consolidation. 

Clinical audit is a quality improvement strategy that provides data on the disparities between current practice and desired performance. It is based on the assumption that practitioners modify their practices when they receive a feedback showing that they are inconsistent with the desirable performance^7^.

A usual clinical audit models is the one adopted by the Joanna Briggs Institute (JBI), which consists of three phases: baseline audit, implementation of best practices, and post-implementation audit^4^.

Although plenty research on good delivery care practices is available, there is a lack of studies on the assessment of the impact of interventions proposing the implementation of this evidence. Aspects such as implementation methodology still need to be better investigated^8^ ^-^ ^9^.

In this sense, this study seeks to answer the following question: does the implementation of evidence-based practices modify normal delivery care?

Thus, this study was proposed to evaluate the impact of the implementation of evidence-based practices in normal delivery care.

## Method

Quasi-experimental, before-and-after intervention study that followed the clinical audit process to implement evidence-based care practices, used by the JBI, composed of the phases: 1) planning and conduction of a baseline audit; 2) evaluation and discussion of results of the baseline audit and educational intervention to implement best practices with the audit team; 3) post-intervention audit, in which the same criteria of the baseline audit are measured and whose objective is to compare the differences between the results of the two audits and to verify the conformity of each audited criterion.

The study was conducted between July 2015 and March 2016 at the Mãe Luzia Women’s Hospital (HMML), a reference public maternity hospital in the state of Amapá, in Macapá, which provides care to women at normal and high obstetric risk. In 2015, the monthly mean of admissions was 650 births (66.5% normal and 33.5% cesarean section) .

This study used data obtained through interviews with health professionals and puerperal women and by consulting the medical records of women attended at the HMML.

In the case of health professionals, it was decided to use the population; 71 professionals were eligible. Of these, 52 met the inclusion criteria, but 10 were not located or did not agree to participate in the study. Thus the remaining 42 professionals participated in the baseline audit (25 nurses and 17 physicians). In addition to these losses, 10 professionals refused or were unavailable in the subsequent phases of the study, resulting in a total of 32 professionals (20 nurses and 12 physicians) who shared in the educational intervention and post-intervention audit. The inclusion criteria of professionals were: obstetric nurse, obstetrician, nursing or medical resident and assistants of normal delivery in the HMML.

The sample size of puerperal women was defined by the prevalence test for before-and-after studies, considering the rate of oxytocin use in the Northern region of Brazil of 22.8%^2^, reducing to 10.5%. Thus, it was estimated that at least 280 women (140 before intervention and 140 after it) were needed to reach an 80% test power and 95% confidence level. The inclusion criteria were: women admitted to the hospital and in the active phase or with overt signs of labor and up to 8 cm of cervical dilatation; women with normal-risk pregnancy and current normal delivery; non-indigenous women, and without special needs.

In both phases, medical records were selected by intentional sampling. In the baseline audit, all hospital discharges of puerperae occurred 30 days before the beginning of this phase were considered. In this moment, 424 medical records were made available and, after checking the inclusion criteria of the puerperae, 291 were selected. In the post-intervention audit, the records of hospital discharge of the puerperae between the second fortnight of February and the first of March of 2016 were included. In this period, 440 medical records were made available, and, after application of inclusion criteria, 264 remained. The following variables were noted: presence of companion, amniotomy, delivery position, oxytocin prescription, oxytocin administration, directed pushing and Kristeller maneuver.

Data collection took place in three phases: a baseline audit was initially carried out (between July and September 2015), with formation of a team and definition of the audit criteria and preliminary evaluation of outcomes and practices used in labor and delivery. The audit team included people involved in the management and care process, called “key professionals”. These were the general manager, the HMML technician and clinical physician, the obstetrics manager, all the physicians, the coordinator of the Nursing team and the head of the Permanent Education Center.

Some practices and maternal outcomes that could indicate the use of the best scientific evidence were adopted as criteria in the audit. These included the increase in the rate of normal delivery, the number of companions chosen by the women, the frequency of the birth position chosen by the women, and upright positions; reduction of amniotomy rate, lithotomy position, intravenous oxytocin, directed pushing and Kristeller maneuver. In this phase, interviews were also carried out with the professionals of the service and with the puerperal women in the rooming-in within 1-2 days postpartum. The questions were related to the presence of companion of the women’s choice, amniotomy, oxytocin, delivery positions, directed pushing, and Kristeller maneuver. Data from the medical records of the puerperal women were also collected.

In the phase 2 (October 2015), an educational intervention called “Seminar of scientific evidence-based care practices to normal delivery” was presented to professionals who participated in the baseline audit. The seminar was held outside the service, lasted 8 hours, and presented the preliminary assessment of the results obtained in the baseline audit and the best practices of delivery care (presence of companion of the choice of the women, upright positions, selective amniotomy and oxytocin, avoidance of Valsalva and Kristeller maneuvers), all of which were discussed with the professionals in the light of scientific evidence. The HMML directors were facilitators in the presentation and discussion of practices. Updated printed material (randomized clinical trials and systematic reviews) were also was made available to all the professionals who shared in the seminar.

However, only 18 (11 nurses and seven physicians) out of the 42 professionals who participated in the baseline audit also participated in this phase. Thus, between October and November of 2015, another strategy was proposed with banners presenting a synthesis of the evidences in the work place of the participants, so as to reach the other professionals. Another 14 professionals (nine nurses and five physicians) participated in this intervention, totaling 32 participants.

Considering that the JBI did not establish the period for development of the post-intervention, the investigators decided to carry it out 60 days after the phase 2. To this end, an audit with the same instruments and criteria applied in the phase 1 was used to identify the application of the practices discussed in the seminar and compare it with the results of the baseline audit.

Data analysis was performed by calculating the absolute and relative frequencies of the categorical variables. Inferential analysis was performed by comparing the results of phases 1 and 3. The chi-square test was used to compare the proportions of the practices identified in the interviews with the puerperae. The Chi-square or Fisher’s exact tests were used to compare the proportions of the practices identified through the consultation of medical records. A methodology for repeated measures with a generalized estimating equation model (GEE) was used to compare the practices identified in the interviews with the professionals in phases 1 and 3^10^.

The study was approved by the Research Ethics Committee of the School of Nursing of the University of São Paulo, under Opinion nº 698.421/2014. The participation of professionals and users of the service was voluntary, after reading, clarifications and signing the Informed Consent Term or Assent Term.

## Results

The comparative analysis between phases 1 and 3 showed that there was an increase of 5.3 percentage points (p.p.) in the normal delivery rate (Table 1).

In the interview with the puerperal women, the post-intervention audit showed that there was a statistically significant increase of the presence of companions of free choice, statistical reduction of amniotomy and of lithotomy position and infusion of oxytocin (Table 2).

**Table 1:** Distribution of deliveries identified by the SAME* of the HMML^†^ 5 months before and 5 months after the intervention - Macapá, AP, Brazil, 2015-2016

Variable	Phase 1		Phase 3	
	N	%	N	%
Type of delivery				
Normal	2.035	63.7	2.257	69.0
Cesarean section	1.160	36.3	1.013	31.0
Total	3.195	100	3.270	100

*SAME: Medical Archive and Statistics Service

†HMML: Mãe Luzia Women’s Hospital

**Table 2:** Practices identified in the interviews with puerperal women in the baseline audit (phase 1) and post-intervention audit (phase 3) and p-values - Macapá, AP, Brazil, 2015-2016

Variable	Puerperae				p-value*
	Phase 1		Phase 3		
	n	%	n	%	
Presence of companion	140		140		
Yes	117	83.6	131	93.6	
No	12	8.6	9	6.4	0.002
It was not of choice	11	7.8	0	0.0	
Realization of amniotomy	140		140		
Yes	74	52.5	50	35.7	0.005
No	66	47.5	90	64.3	
Choice of birth position	140		140		
Yes	79	56.4	84	60.0	
No	61	43.6	56	40.0	0.545
Position at delivery	140		140		
Lithotomy	105	75.0	71	50.7	<0.001
Upright/Squat	15	10.7	59	42.1	
Lateral	12	8.6	7	5.0	
Four supports	8	5.7	3	2.2	
Use of oxytocin	140		140		
Yes	84	60.0	60	42.9	0.004
No	56	40.0	80	57.1	
Directed pushing incentive	140		140		
Yes	111	79.3	70	50.0	<0.001
No	29	20.7	70	50.0	
Kristeller maneuver	140		140		
Yes	29	20.7	14	10.0	
No	111	79.3	126	90.0	0.013

*Chi-squared test

The interviews with the professionals revealed a statistical decrease of oxytocin prescription or administration after the intervention. No statistical differences were seen in the other practices (Table 3).

**Table 3:** Practices identified in the interviews with professionals in the baseline audit (phase 1) and post-intervention audit (phase 3) and p-values - Macapá, AP, Brazil, 2015-2016

Variable	Professionals				p-value*
	Phase 1		Phase 3		
	n	%	n	%	
Allows the presence of companion	42		32		
Always/most of the times	41	97.6	32	100	0.325
Rarely/never	1	2.4	0	0.0	
Performs amniotomy	42		32		
Always/most of the times	12	28.6	6	18.7	0.320
Rarely/never	30	71.4	26	81.3	
Allows the choice of delivery position	42		32		
Always/most of the times	33	78.6	27	84.4	0.521
Rarely/never	9	21.4	5	15.6	
Recommends lithotomy position	42		32		
Always/most of the times	19	45.2	11	34.4	0.343
Rarely/never	23	54.8	21	65.6	
Recommends lateral position	42		32		
Always/most of the times	22	52.4	18	56.3	0.741
Rarely/never	20	47.6	14	43.7	
Recommended upright position	42		32		
Always/most of the times	17	40.5	13	40.6	0.990
Rarely/never	25	59.5	19	59.4	
Prescribes/administers oxytocin	42		32		
Always/most of the times	19	45.2	5	15.6	0.005
Rarely/never	23	54.8	27	84.4	
Guides/encourages directed pushing	42		32		
Always/most of the times	18	42.9	7	21.9	0.054
Rarely/never	24	57.1	25	78.1	
Performs/encourages Kristeller maneuver	42		32		
Always/most of the times	2	4.8	0	0.0	0.716
Rarely/never	40	95.2	32	100	

* GEE (Generalized estimating equation model)

In the analysis of medical records, there were a large number of records in the information topic. After the intervention, there was a statistically significant difference in the performance of amniotomy and in the use of lateral position during delivery (Table 4).

**Table 4:** Practices identified in the medical records in the baseline audit (phase 1) and post-intervention audit (phase 3) and p-values - Macapá, AP, Brazil, 2015-2016

Variable	Medical record				p-value
	Phase 1		Phase 3		
	N	%	n	%	
Presence of companion	291		264		
Yes	13	4.5	44	16.7	1.000*
No	0	0.0	3	1.1	
No record	278	95.5	217	82.2	
Realization of amniotomy	291		264		
Yes	130	44.7	40	15.2	<0.001^†^
No	141	48.4	178	67.4	
No record	20	6.9	46	17.4	
Position at delivery	291		264		
Lithotomy	74	25.4	63	23.9	0.013*
Upright/Squat	20	6.9	24	9.1	
Lateral	31	10.6	9	3.4	
Four supports	4	1.4	5	1.9	
No record	162	55.7	163	61.7	
Oxytocin prescription	291		264		
Yes	151	51.9	131	49.6	0.593^†^
No	140	48.1	133	50.4	
Oxytocin administration	291		264		
Yes	150	51.5	122	46.2	0.209^†^
No	141	48.5	142	53.8	
Kristeller maneuver	291		264		
Yes	2	0.7	2	0.8	1.000^†^
No	0	0.0	0	0.0	
No record	289	99.3	262	99.2	

*Fisher’s exact Test

†Chi-square test

## Discussion

This quasi-experimental, before-and-after intervention study was based on the clinical audit model and sought to assess the impact of the implementation of evidence-based practices on normal delivery care. Few studies have explored this subject in the obstetric area despite its great clinical and academic relevance.

The care model adopted in Brazilian hospitals results in the exposure of women, especially those with habitual obstetric risk, to unnecessary interventions that lack evidence to justify their use^2^. Thus, the protocol of this research defined some practices and maternal outcomes that represent the use of the best evidence in childbirth care recommended by the WHO. In the HMML, such changes were fundamental in view of the framework found in the baseline audit, that is, high rates of interventions without scientific recommendation or even considered iatrogenic.

Although this study has resulted in improved clinical practice, scientific evidence-based childbirth care practices has not been fully implemented, possibly because this is a complex and continuous process that involves changes and overcoming of barriers at the individual and institutional levels, as it has been indicated by other researchers^4^ ^,^ ^11^.

The scientific literature points out several factors that hinder the implementation of evidence-based clinical practice. At the organizational level, the main barriers are lack of time, inadequate facilities and lack of support^12^. In the hospital where this research was conducted, workload of professionals was detected, as well as a small number of beds inconsistent with the high demand of deliveries, and lack of physical infrastructure. The implementation of evidence could have been more successful if there were an organizational context that supported evidence-based practice.

At the individual level, barriers include lack of knowledge about research methods and results and negative attitude towards evidence-based practice^12^. Added to this is the resistance of some health professionals who cannot break up with the current paradigm of childbirth care^13^, probably because they were trained in a time before the launching of humanization policies and evidence-based practice. Moreover, medical education does not yet focus on the training of professionals to provide comprehensive, quality and humanized care, but it is rather inclined to reproduce the use of interventionist practices^13^ ^-^ ^14^.

The competition imposed by other health priorities, the scarcity of resources, the lack of motivation to implement and sustain the changes in the practice of care and the ineffective dissemination of the results are factors that contribute to the resumption of the previous practice after a research intervention^11^.

There are a large number of strategies that can contribute to an effective implementation of changes in the clinical practice. These are based on different theories about human behavior, professional change and organizational performance. The literature suggests that real and sustainable changes can be achieved by combining these different approaches^15^. 

After the educational intervention, there was an increase of 5.3 p.p. in the normal delivery rate, possibly stimulated by discussion of the implementation of evidence-based practices in the seminar. However, because of the limitations of the study design, it is not possible to state that this was the only determinant for this change. Additional data, such as the risk situation of women during labor progression and indications to perform cesarean section, were not analyzed. In any case, it was noticed that more professionals began to adopt practices that contribute to the viability of normal delivery, including the presence of companions, and reduction of amniotomy and of infusion of oxytocin.

The post-intervention audit revealed a significant increase in the number of women who had companions of their choice during labor and delivery. Individual support has beneficial clinical effects to women and newborns, as indicated by scientific evidence. Such support results in shorter labor, greater chance of spontaneous delivery, less need for analgesia, fewer newborns with an Apgar score in the fifth minute and fewer reports of dissatisfaction with childbirth, which should be guaranteed for every woman^16^.

In Brazil, the presence of a companion of the woman’s free choice during childbirth has been guaranteed, for more than 10 years, by Federal Law nº 11,108, of April 7, 2005^17^. Furthermore, the National Agency for Supplementary Health concluded, through Normative Resolution 387, that obstetric care in the private sector should cover the expenses of the companion, including proper clothing, lodging and meals, regardless of health insurance^18^.

The National Guideline on Childbirth also strengthens the recommendation that women have companions of their choice during labor and delivery, not invalidating the support given by persons that are outside the women’s social network^1^. This is a demonstrably useful practice and should be encouraged, since it contributes to the humanization of care and reduction of unnecessary obstetric interventions^3^.

Nevertheless, it is important to point out that, in recent years, the legitimizing of the respect for this right has faced difficulties in Brazil, mainly because of the professionals’ resistance to the presence of companions, lack of physical structure, lack of human and material resources in health institutions and lack of institutional support and guidelines for implementation of the Law on Companions^14^.

In the HMML, the structure of the old obstetric center, the current CPN, did not offer physical conditions to satisfactorily host the women, their companions and the professionals. In 2014, this structure has undergone changes to adapt the environment as established in the current policy on assistance for women, of the Cegonha Network/MOH. Thus, the changes in space associated with the educational intervention carried out in this study may justify the increase in the presence of companions of the women’s choice during delivery. 

The decrease in the rate of amniotomy after the intervention was an unexpected finding because, in the HMML, this practice was performed in a way associated to oxytocin infusion under the justification of reducing the length of stay of the women in the delivery center, what was necessary due to the high demand in the service. Amniotomy before full cervical dilatation is often used to accelerate labor, but the effectiveness of this intervention has not been proven and remains the subject of debate and investigation.

Two systematic reviews without meta-analysis were conducted to evaluate the effectiveness of active labor management. The first one, with 5,390 women, evaluated whether this type of maneuvering reduced the number of cesareans in normal-risk gestations and if it improved the satisfaction of the women. Practices included routine amniotomy, oxytocin infusion, and individual support in labor. The authors concluded that active management is associated with a small reduction in the cesarean rate, but it is highly prescriptive and interventional. More studies are therefore needed to evaluate the acceptability of this management by the parturient^19^.

The second review concluded that there is no evidence that amniotomy is associated with shortened cervical dilatation, cesarean section rate, maternal satisfaction, and Apgar score at the fifth minute^20^. Therefore, this procedure should not be routinely adopted as part of parturient care.

As for delivery position, there was a significant reduction of the use of lithotomy and a substantial increase in the adoption of upright positions after the intervention. This finding may be justified by the greater participation of obstetrical nurses in the seminar. It was also observed that, in the daily practice, these professionals started to guide women more often regarding the different delivery positions and giving them the possibility of free choice.

Our results are in line with the findings of a study carried out in São Paulo (Brazil) in which the authors observed that, after an educational intervention with the professionals of a maternity ward, upright positions were adopted by all parturient women and lithotomy was no longer used, a finding supported by statistical significance (p = 0.001)^21^.

In humanized care, women are encouraged to use their freedom to choose their position at labor and delivery. However, in Brazil, the lithotomy position continues to be used during the expulsive period by the majority of parturient^2^.

Study shows that, when women adopt vertical positions, the physical and psychological benefits include shorter duration of labor, fewer interventions and less severe pain, and greater satisfaction with childbirth^22^. A systematic review concluded that it is not yet possible to estimate the risks and benefits of different birth positions because of the poor methodological quality of the available studies. Thus, every woman should have the possibility to choose the position she wants in childbirth. However, a substantial reduction of attended deliveries, episiotomy, and increase in second-degree perineal lacerations are observed in the upright position, without epidural anesthesia^23^.

According to the puerperal women, the post-intervention audit also revealed a statistical reduction in the number of women subjected to oxytocin infusion during labor. Such an outcome can be explained by the impact of the seminar (in line with the advice that oxytocin infusion should not be a routine practice) and the introduction of other practices in childbirth care, such as encouragement to walking, warm baths and an the opportunity to choose the delivery position, in particular the upright ones, which make it possible to accelerate labor.

A systematic review evaluated the use of oxytocin to accelerate the slow progression of dilatation *versus* the reductions of cesarean rates and maternal and fetal morbidity and found no statistical difference in the frequency of cesarean sections (p = 0.88) or in adverse maternal and neonatal outcomes (p = 1.02) when comparing to late use or non-use of the hormone. In contrast, it was observed that the early use of the drug resulted in uterine hyperstimulation associated with fetal cardiac changes and reduction of labor in about 2 hours^24^. Thus, infusion of oxytocin during the period of dilatation should be restricted to specific situations such as failures in the progress of labor, in which there is a need for correction of uterine dynamics^3^. Thus, the results achieved after the educational intervention seem to favor the relationship between the care practices and the scientific evidence in the service where this research was conducted.

Directed pushing and Kristeller maneuver were also significantly reduced after the educational intervention. When comparing the results of the interviewed mothers with those of the professionals, the data on these practices reveal partial agreement, since only directed pushing had a statistically significant decrease.

Regardless of complete cervical dilatation, stimulating the parturient to force, preventing her from obeying her own impulses, thus disrespecting the physiology of childbirth, is a frequent practice in maternities^3^. The National Guideline on Parturition Care recommends spontaneous pushing during the expulsive period in women without analgesia, and avoidance of directed pushing^1^. 

A recent literature review that assessed maternal and neonatal morbidity associated with the type of pushing used during the expulsive period found that the groups did not differ in perineal lesions, episiotomies or type of delivery. Only one study found a higher Apgar score in the fifth minute and better umbilical artery pH in the spontaneous pushing group. The study concluded that the low methodological quality of the studies and the differences between the protocols do not justify recommendations on any type of pushing^25^.

Professionals justify their guiding long and directed pushing with the purpose of shortening the expulsive period^3^. However, the systematic review that investigated the interference of directed pushing in the expulsive period concluded that this action resulted in no effect in the length of this period and in the rates of perineal trauma when compared to spontaneous pushing^26^. Therefore, the woman should be encouraged to follow her own impulses.

Regarding the Kristeller maneuver, an observational study was performed in Egypt with 8,097 women undergoing normal labor to verify the effects of this maneuver. The authors found that, despite the shorter duration of the second period, there was a significant increase in the risk of severe perineal lacerations, uterine rupture, dyspareunia and urinary incontinence 6 months postpartum. In the NB, there were shoulder dystocia, increased risk of Apgar scores below seven in the fifth minute, fetal sequelae such as hypoperfusion and cerebral palsy^27^.

The practices and maternal outcomes identified in the interviews with professionals showed that, after the intervention, all of them reported “always or most of the times” to allow women to choose their companions, but this result had was not statistically significant, because almost all professionals already did so before the intervention. Similar results were found in the interviews of the women. Thus, it can be inferred that the educational intervention improved this practice, ratifying the maintenance of scientific evidence.

Regarding amniotomy, although there was a decrease in the number of professionals who reported they “always or most of the times” adopt this procedure, the result was not significant. This finding differs from that obtained in the interviews with the puerperal women, probably due to the small number of professionals interviewed.

It is worth mentioning that, because amnioscopes were not available in the studied scenario, amniotomy is used as a method to assess the appearance of amniotic fluid, which may have contributed to the maintenance of high rates and the difficulty in changing the care practice.

Regarding the delivery position, different from the women’s reports, there was no significant difference in the reduction of the lithotomy rate. It was also verified that the interviews of both professionals and puerperal women revealed no significant difference in the possibility of choosing the delivery position by the parturient, showing that the decision of this aspect is still centered on the professionals.

An observational study conducted in Nigeria, which aimed to identify the relationship between delivery positions and perineal trauma, revealed high lithotomy rates. Women who adopted this position (85%) reported it as of no help for the progress of labor, but that they had been given no choice, due to the imposition of professionals. The midwives had used this practice in 98% of the births, justifying the conduct with the argument that this is a condition imposed by the institution. Routine use of the lithotomy position is a reflection of the medical culture incorporated into these hospitals, and midwives are educated and trained to assist women in this position^28^.

The most effective care is the one in which the parturient is the central figure and her needs are valued at the expense of the demands of professionals or institutions.

Prescription/administration of oxytocin at delivery had a statistical decrease after the intervention. It should be noted that, in the years prior to the intervention, this drug was routinely prescribed and associated with amniotomy for the active management of labor, especially by medical professionals, under the justification of reducing the time of the women in the obstetric center in view of the high demand in the service.

Administration of oxytocin, whether or not associated with early amniotomy, should not be routinely performed in cases of labor with good progression^1^.

A systematic review compared the low initial dose versus high initial dose of oxytocin and indicated that the higher dosage of the drug significantly reduced the duration of delivery (± 3.5h) and cesarean rates, and increased vaginal delivery. The study concluded that there is insufficient evidence to recommend the use of high doses of oxytocin in women with slow progression of labor, and recommended further research to evaluate this effect^29^.

There was a non-significant reduction in orienting or encouraging directed pushing and Kristeller maneuver after the educational intervention. The reduction of these interventions, although satisfactory, did not allow the audit criterion initially defined to be achieved. The persistence of directed pushing and Kristeller maneuver how’s that there is still resistance from the part of professionals to change the interventionist care model which is based on previous beliefs or experiences. Since the studied maternity is a teaching hospital, this scenario is even more worrying, as it may result in the perpetuation of this delivery model.

Since the planning of this study, difficulties to retrieve information from medical records were expected, especially with respect to some practices that, although performed, are often hidden, especially for the risk that they represent to the parturient women and the newborns, such as directed pushing and Kristeller maneuvering. Other practices such as oxytocin use, amniotomy, birth position and presence of companion were chosen because they are important indicators in the obstetric area and, in general, recorded in medical charts. For some of these variables, the quantity and quality of the records were deficient, limiting the discussion of the findings of this study.

After the educational intervention, the record of the presence of companions increased, although without statistical significance, probably because this information was still absent in more than 90% of the medical records. On the other hand, all the professionals interviewed in the phase 3 reported that they “always or most of the times” allow the presence of companions, and more than 90% of the puerperae reported having been allowed to have a companion during labor.

As for the practice of amniotomy, it was found that there was a decrease of records in the medical charts after the intervention, with a significant difference. This result is similar to the reports of puerperae, but differs from those of the professionals. It is worth clarifying that only when the amniotic sac was described as intact in the medical record at the moment of admission without reference to artificial rupture until delivery is that non-realization of amniotomy was noted. Furthermore, in the post-intervention audit, the missing records of this practice more than doubled compared to the baseline audit.

The registration of membrane integrity is an important factor in the evolution of labor, in contrast with the negative repercussions of artificial rupture on maternal and fetal health. There is evidence that undesirable effects result from this intervention, including increased early deceleration of fetal heart rate and a higher risk of fetal and puerperal infection^1^.Thus, it is essential to encourage the recording of this information in the medical record.

Regarding the positions adopted in the expulsive period, the records in the charts showed that, after the educational intervention, there was a decrease in the lithotomy position, while upright/squatting position increased, with a significant difference. This finding corroborates the reports of the puerperal women, but not those of the professionals, probably, due to the small number of professionals interviewed.

The records in the medical records concerning the prescription of oxytocin during labor revealed that this practice was proportionally reduced after the educational intervention, but without statistical difference. In the post-intervention audit, it was found that the use of oxytocin in the period of dilatation was less frequency in all sources of data audited, i.e., interviews with puerperal women, professionals and medical records. It should be noted that for this variable, the information was available in the medical records.

These findings are in line with a Palestinian study that used the methodology to implement better evidence in normal childbirth care to investigate possible changes in practices adopted by professionals. The authors demonstrated a lower frequency of some important practices, including liberal use of oxytocin and artificial rupture of membranes after the intervention, with statistically significant difference^30^.

The absence of pre-defined audit criteria in the JBI for the implemented practices was an important limitation in this study. Other limitations were the high frequency of missing data on the practices in the medical records, the non-randomized collection of puerperae to be interviewed, and the difficulty to recruit professionals.

Our findings not only bring contributions to the knowledge of professionals who assist labor and delivery, but also eliminate empirical, routine and unnecessary care measures for parturient women and improve clinical practice. 

## Conclusion

Our results allow us to infer that the methodology of implementation of scientific evidence improved some obstetric practices and maternal outcomes. There was an increase in the rate of normal delivery. In the other outcomes, the improvements found varied according to the information source. From the perspective of the puerperae, there was a significant increase of free choice of companions during labor and the upright/squatting position, a significant reduction of amniotomy, lithotomy position, oxytocin infusion, directed pushing and Kristeller maneuvering. From the perspective of professionals, the practice that presented statistical decrease was prescription/administration of oxytocin during labor. The analysis of the medical charts showed a statistical reduction of amniotomy and lithotomy, with a consequent increase of upright/squatting positions. 
